# Deprivation and prognosis in patients with pulmonary arterial hypertension: missing the effect of deprivation on a rare disease?

**DOI:** 10.1183/13993003.02334-2019

**Published:** 2020-08-13

**Authors:** Eleni Sofianopoulou, Colin Church, Gerry Coghlan, Luke Howard, Martin Johnson, David G. Kiely, Allan Lawrie, James Lordan, Martin R. Wilkins, Stephen J. Wort, Nicholas W. Morrell, Mark R. Toshner

**Affiliations:** 1 Dept of Public Health and Primary Care, Cardiovascular Epidemiology Unit, University of Cambridge, Cambridge, UK; 2 Golden Jubilee National Hospital, Glasgow, UK; 3 Royal Free Hospital, London, UK; 4 Imperial College London, London, UK; 5 Sheffield Pulmonary Vascular Disease Unit, Royal Hallamshire Hospital, Sheffield, UK; 6 Dept of Infection, Immunity and Cardiovascular Disease, University of Sheffield, Sheffield, UK; 7 University of Newcastle, Newcastle, UK; 8 Royal Brompton Hospital, London, UK; 9 Dept of Medicine, University of Cambridge, Cambridge, UK; 10 NIHR BioResource - Rare Diseases, Cambridge, UK; 11 Supervision of this work was shared by E. Sofianopoulou and M.R. Toshner

## Abstract

In this journal, Pellino *et al.* [1] presented a survival analysis to assess how deprivation affects prognosis in patients with pulmonary arterial hypertension (PAH). Their conclusions were that social deprivation is not a significant referral barrier or prognostic factor for idiopathic (I)PAH or heritable (H)PAH in Scotland. This may appear surprising, given the wider context of literature describing outcomes stratified by social deprivation. The authors were thorough on using both the address at time of diagnosis and at time of censoring to assign deprivation scores and compare the two, finding no significant differences between the two approaches. They also compared deprivation assigned to PAH cases to expected deprivation based on Scottish citizenry as a whole, and found that PAH patients are more socially deprived than expected. Finally, they used the same survival univariate analysis adjusting for age and sex to assess how several clinical variables are associated with prognosis.

## Abstract 2

**No association was found between deprivation and mortality for PAH patients in England and Wales. The association found between risk stratification at baseline and deprivation suggests that the issue of deprivation and outcomes in PAH may be more nuanced.** https://bit.ly/2y8WgqB

*To the Editor*:

In this journal, Pellino *et al.* [1] presented a survival analysis to assess how deprivation affects prognosis in patients with pulmonary arterial hypertension (PAH). Their conclusions were that social deprivation is not a significant referral barrier or prognostic factor for idiopathic (I)PAH or heritable (H)PAH in Scotland. This may appear surprising, given the wider context of literature describing outcomes stratified by social deprivation. The authors were thorough on using both the address at time of diagnosis and at time of censoring to assign deprivation scores and compare the two, finding no significant differences between the two approaches. They also compared deprivation assigned to PAH cases to expected deprivation based on Scottish citizenry as a whole, and found that PAH patients are more socially deprived than expected. Finally, they used the same survival univariate analysis adjusting for age and sex to assess how several clinical variables are associated with prognosis.

The ongoing National Cohort Study of Idiopathic and Heritable PAH collects data from all of the UK and offers an opportunity to test the relationship between deprivation and outcomes in PAH in a second population that is geographically distinct but in many other ways highly analogous to that studied by Pellino *et al.* [1]. The study collects data from a contemporaneous cohort in all of the specialist PAH centres in the UK. Therefore, we were able to assess mortality in relation to deprivation in 270 PAH patients living in England and Wales, residing in the same addresses since diagnosis. Our patients were recruited in the UK PAH cohort from 1 January, 2014 and followed-up until 28 February, 2019. 36 (13%) participants died and five (2%) were transplanted, resulting in 1-, 3- and 5-year transplant-free survival estimates of 96%, 86% and 73% for incident patients (n=146) and 100%, 100% and 89% for prevalent patients (n=124), respectively. Pellino *et al.* [1] captured deprivation using the Scottish Index of Multiple Deprivation (IMD), which is a composite index comprising of sub-indices (*e.g.* income, education, employment, health, access to services, crime). In the UK, IMD is not comparable between England, Scotland and Wales, so for our UK cohort we accessed an adjusted IMD index for use across the UK [2], which consists of the three main domains of IMD: income, employment and educational deprivation. In addition to the area level deprivation captured *via* the UK-IMD, the primary survival model was adjusted for age at diagnosis and sex, to be in line with the adjustment used by Pellino *et al.* [1], fitting Cox proportional hazards regression models. Cox proportional hazards regression models and survival curves were fitted allowing for left truncation arising from the interval between diagnosis of prevalent cases and enrolment. Prevalent patients were only included in the risk set from the time of study entry and were excluded if they entered the study >10 years after diagnosis. We stratified the analysis by centre, due to the multicentre design of our study. In the extended model, we further adjusted for smoking, prevalent/incident status and risk profiles (low/intermediate/high) at the time of diagnosis, calculated using the abbreviated 2015 European Society of Cardiology (ESC)/European Respiratory Society (ERS) risk stratification strategy [3]. According to data availability, the risk score groups were estimated using the World Health Organization functional class, right atrial pressure, cardiac index and mixed venous oxygen saturation, having significant difference (p=0.02) on survival (figure 1), as expected.

**FIGURE 1:**
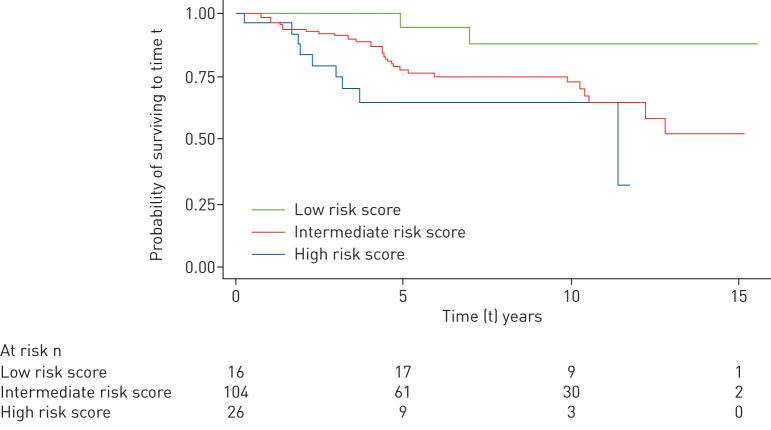
Transplant-free survival in relation to risk profile (Kaplan–Meier estimates), at the time of diagnosis (n=270).

The study participants are more likely to be women (68%) with mean±sd age at diagnosis 51±16 years. The pulmonary haemodynamic characteristics of the study participants were: 1) right atrial pressure 9±5 mmHg, 2) mean pulmonary arterial pressure, 53±14 mmHg, 3) cardiac index 2±0.6 L·min^−1^·m^−2^, 4) cardiac output 4±1.2 L·min^−1^, 5) pulmonary vascular resistance 12±5 WU and 6) mixed venous oxygen saturation, 64±8%. We found no association between mortality and classes of area deprivation, for both the primary and adjusted model. As an example, adjusted hazard ratio (HR) was 0.63 (95% CI 0.18–2.16) (p=0.458) for most deprived (quintile 5) areas compared to least deprived (quintile 1) areas. To examine this further, we investigated area deprivation as predictor of risk profiles, fitting multinomial regression models. We found an association between risk profiles and area deprivation, while adjusting for the same variables as above. Living within the more deprived areas (quintiles 5, 4 and 3) was associated with higher PAH risk score at baseline, compared to the least deprived areas (quintile 1), therefore increasing the likelihood of being diagnosed within the ESC/ERS high-risk clinical prediction category. Testing for the overall effect of deprivation to risk profiles, we found a statistically significant predictor effect (p=0.034, Wald-test). The relative risk ratio changing from least (quintile 1) to most (quintile 5) deprived area is 15.01 (95% CI 1.96–114.96) (p=0.009), for being classed as high risk profile *versus* low risk profile. This was 14.59 (95% CI 2.22–95.9) (p=0.005) and 9.75 (95% CI 1.42–67.06) (p=0.021) for quintiles 4 and 3, respectively. In other words, we found that the expected risk for being in high-risk class is higher for subjects who live in the most deprived areas. The baseline risk class was also found to be a predictor of mortality in this survival analysis (adjusted HR 6.71, 95% CI 1.22–36.87) (p=0.028), in line with the literature [3–5].

The notion that deprivation is associated with increased mortality and morbidity for the most deprived compared with the most affluent, is confirmed by many studies and, therefore, has been enshrined in health studies, theories and policies [6–9]. The main aspects of deprivation are income, employment and education that can be collected at area-level and individual level or, preferably, both. The choice for using a composite index or its subdomains, or individual-level deprivation, is driven by the research question and availability of deprivation indicators. Studies have faced numerous challenges in terms of using a composite area-level deprivation index. The data required are based on coverage of routinely collected data, which can vary between countries, using also a weighting score that depends on the priorities set by each country, making these indices not comparable. Usually, though, the highest weights are given to income, employment and educational deprivation and, therefore, it is possible the composite index is similar to indices that are based on any of these traditional deprivation markers. Still, we recommend not to assume that any differences would have no impact on the results, especially when using small datasets as the models can be too sensitive.

Even when such an index is used for the intended geographical area it was created for, the geographical distributions of sub-indices may have opposing directions, introducing error on the overall measure of deprivation. For instance, a high deprivation score should reflect both areas of low income and difficulty in accessing healthcare, with the latter often being measured as distance to primary healthcare centre. Patients who live near city centres, which tend to have higher income deprivation, may not have a long distance to access a primary care centre, while the opposite holds for wealthier populations that may live outside the city centre. Thus, the estimated composite deprivation can be compromised. Even the traditional indicators of income and education deprivation may vary substantially across areas [10]. Any differences can be understood by mapping the individual domains, which is important when considering how such data is used in analyses.

Composite IMD indices, such as the Scottish IMD used by Pellino *et al.* [1], have a health subdomain calculated based on standardised mortality ratio among other variables, so PAH mortality may be captured in some degree by this sub-domain. Therefore, ideally, the health sub-domain should not be included when deprivation is examined in relation to mortality. Finally, we would argue that even when prognosis is not associated with deprivation, this cannot directly lead to the interpretation that prognosis is not affected by access to care and referrals. Deprivation is linked to several parameters that affect health (*i.e.* diet, air quality) other than just equity on access to health services.

A notable feature of the UK National Health Service specialised commissioning structure for PAH is the equity of access to all licensed treatments to all patients, once they have been referred to the centres. A possible interpretation of our results and those reported by Pellino *et al.* [1] is therefore that deprivation acts as a barrier to referral but once in the specialised system, the equity of treatment availability diminishes the subsequent effect on mortality. We feel it cannot be ignored that, unlike previous work that showed a significant effect of deprivation on mortality [11], both UK studies of similar sample sizes have failed to demonstrate an association with mortality and in health systems with more equal access to licensed treatment.

There are some limitations to this work. It is common for rare disease projects to analyse hundreds of cases, compared to thousands or millions in common diseases, for the same or similar research questions [12, 13], leading to the need to adopt refined datasets and analytical methods. The introduction of measurement error when using a deprivation index is likely to affect small datasets disproportionately and needs to be used with caution when conducting sensitivity analysis. Lastly, we are unable to quantify the effect of patients potentially not surviving to first admission to a tertiary PH care centre, and this drop out is theoretically more significant, specifically because of the association we find with baseline risk stratification.

Overall, the study by Pellino *et al.* [1] is an important piece in the PAH aetiology puzzle, and the results of our study are in line with their lack of association between mortality and social deprivation; although we would caution that both studies are of modest size. At the same time, Pellino *et al.* [1] found that PAH patients are more socially deprived than expected, based on Scottish citizenry as a whole. This alludes that deprivation status may have an association with PAH rather than it being a random finding. Our study adds further to the literature by demonstrating that higher area deprivation is associated with a worse risk profile at baseline, which in turn is a predictor of worse survival rates, suggesting that the issue of social deprivation and outcomes in PAH may be more nuanced. Examining both primary healthcare and hospital data in PAH could provide evidence on whether in deprived areas delays on PAH diagnosis are associated with the patient taking longer to seek help and the time required for tests related to the diagnosis, compared to least deprived areas.

## Shareable PDF

10.1183/13993003.02334-2019.Shareable1 This one-page PDF can be shared freely online.
Shareable PDF ERJ-02334-2019.Shareable

## Supplementary Material

ERJ-02334-2019.Shareable.pdf
