# Supplementary material for: Deprivation and prognosis in patients with pulmonary arterial hypertension: missing the effect of deprivation on a rare disease?
Source: Eur Respir J. 2020 Aug 13;56(2):1902334. doi: 10.1183/13993003.02334-2019 (PMC7424115; doi:10.1183/13993003.02334-2019)
Supplement: Supplementary file 1 [file ERJ-02334-2019.Shareable.pdf]

AGORA
RESEARCH LETTER

SHAREABLE PDF

Deprivation and prognosis in patients with pulmonary arterial hypertension: missing the effect of deprivation on a rare disease?

Eleni Sofianopoulou[1,11], Colin Church[2], Gerry Coghlan[3], Luke Howard[4], Martin Johnson[2], David G. Kiely[5], Allan Lawrie[6], James Lordan[7], Martin R. Wilkins[4], Stephen J. Wort[4,8], Nicholas W. Morrell[9,10] and Mark R. Toshner[9,11]

Affiliations: [1]Dept of Public Health and Primary Care, Cardiovascular Epidemiology Unit, University of Cambridge, Cambridge, UK. [2]Golden Jubilee National Hospital, Glasgow, UK. [3]Royal Free Hospital, London, UK. [4]Imperial College London, London, UK. [5]Sheffield Pulmonary Vascular Disease Unit, Royal Hallamshire Hospital, Sheffield, UK. [6]Dept of Infection, Immunity and Cardiovascular Disease, University of Sheffield, Sheffield, UK. [7]University of Newcastle, Newcastle, UK. [8]Royal Brompton Hospital, London, UK. [9]Dept of Medicine, University of Cambridge, Cambridge, UK. [10]NIHR BioResource - Rare Diseases, Cambridge, UK. [11]Supervision of this work was shared by E. Sofianopoulou and M.R. Toshner.

Correspondence: Eleni Sofianopoulou, University of Cambridge, Dept of Public Health and Primary Care, Wort's Causeway, Strangeways Research Laboratory, Cambridge CB1 8RN, UK. E-mail: es630@medschl.cam.ac.uk

@ERSpublications
No association was found between deprivation and mortality for PAH patients in England and Wales. The association found between risk stratification at baseline and deprivation suggests that the issue of deprivation and outcomes in PAH may be more nuanced. https://bit.ly/2y8WgqB

Cite this article as: Sofianopoulou E, Church C, Coghlan G, *et al.* Deprivation and prognosis in patients with pulmonary arterial hypertension: missing the effect of deprivation on a rare disease?. *Eur Respir J* 2020; 56: 1902334 [https://doi.org/10.1183/13993003.02334-2019].

This single-page version can be shared freely online.

To the Editor:

In this journal, Pellino *et al.* [1] presented a survival analysis to assess how deprivation affects prognosis in patients with pulmonary arterial hypertension (PAH). Their conclusions were that social deprivation is not a significant referral barrier or prognostic factor for idiopathic (I)PAH or heritable (H)PAH in Scotland. This may appear surprising, given the wider context of literature describing outcomes stratified by social deprivation. The authors were thorough on using both the address at time of diagnosis and at time of censoring to assign deprivation scores and compare the two, finding no significant differences between the two approaches. They also compared deprivation assigned to PAH cases to expected deprivation based on Scottish citizenry as a whole, and found that PAH patients are more socially deprived than expected. Finally, they used the same survival univariate analysis adjusting for age and sex to assess how several clinical variables are associated with prognosis.

Copyright ©ERS 2020.. This version is distributed under the terms of the Creative Commons Attribution Licence 4.0.
